# Drug-induced gingival hyperplasia: a retrospective study using spontaneous reporting system databases

**DOI:** 10.1186/s40780-017-0088-5

**Published:** 2017-07-19

**Authors:** Haruna Hatahira, Junko Abe, Yuuki Hane, Toshinobu Matsui, Sayaka Sasaoka, Yumi Motooka, Shiori Hasegawa, Akiho Fukuda, Misa Naganuma, Tomofumi Ohmori, Yasutomi Kinosada, Mitsuhiro Nakamura

**Affiliations:** 1 Laboratory of Drug Informatics, Gifu Pharmaceutical University, 1-25-4 Daigaku-nishi, Gifu, 501-1196 Japan; 2 Medical Database Co., Ltd, 3-11-10 Higashi, Shibuya-ku, Tokyo, 150-0011 Japan; 3 Ace Pharmacy, Seiyou Trading Co., Ltd, 1343 Funatsuke Nakashiro, Yoro-cho, Gifu, 503-1382 Japan; 4 United Graduate School of Drug Discovery and Medical Information Sciences, Gifu University, 1-1 Yanagido, Gifu, 501-1194 Japan

**Keywords:** Drug-induced gingival hyperplasia (DIGH), JADER, FAERS, Time-to-onset analysis, Association rule mining technique, Spontaneous reporting system

## Abstract

**Background:**

Drug-induced gingival hyperplasia (DIGH) causes problems with chewing, aesthetics, and pronunciation, and leads to the deterioration of the patient’s quality of life (QOL). Thus, the aim of this study was to evaluate the incidence of DIGH using spontaneous reporting system (SRS) databases.

**Methods:**

We analyzed reports of DIGH from SRS databases and calculated the reporting odds ratios (RORs) of suspected drugs (immunosuppressants, calcium channel blockers, and anticonvulsants). The SRS databases used were the US Food and Drug Administration (FDA) Adverse Event Reporting System (FAERS) and the Japanese Adverse Drug Event Report (JADER) database. With the data, we evaluated the time-to-onset profile and the hazard type using the Weibull shape parameter (WSP). Furthermore, we used the association rule mining technique to discover undetected relationships such as possible risk factors.

**Results:**

The FAERS contained 5,821,716 reports. The RORs (95% confidence interval: CI) for cyclosporine, everolimus, sirolimus, mycophenolate mofetil, amlodipine, nifedipine, carbamazepine, clobazam, levetiracetam, phenobarbital, phenytoin, primidone, topiramate, and valproic acid, were 39.4 (95% CI: 30.3–51.2), 4.2 (1.7–10.0), 6.6 (2.5–17.7), 13.1 (7.2–23.2), 94.8 (80.0–112.9), 57.9 (35.7–94.0), 15.1 (10.3–22.3), 65.4 (33.8–126.7), 6.5 (3.6–11.8), 19.7 (8.8–44.0), 65.4 (52.4–82.9), 56.5 (21.1–151.7), 2.9 (1.1–7.7), and 17.5 (12.6–24.4), respectively. The JADER database contained 430,587 reports. The median time-to-onset of gingival hyperplasia values for immunosuppressants, calcium channel blockers, and anticonvulsants use were 71, 262, and 37 days, respectively. Furthermore, the 95% CI of the WSP β for anticonvulsants was over and excluded 1, which meant that they were wear-out failure type.

**Conclusions:**

Our results suggest that DIGH monitoring of patients administered immunosuppressants, calcium channel blockers, or anticonvulsants is important. We demonstrated the potential risk of DIGH following the long-term use of calcium channel blocker over approximately 260 days. Based on the results of the association rule mining approach, patients with intellectual disability who are administered phenytoin should be monitored carefully. We recommend that patients who experience symptoms related to DIGH should be closely monitored.

## Background

Drug-induced gingival hyperplasia (DIGH) is a periodontal side effects of certain drugs, causing swelling, bleeding, and problems with chewing, aesthetics, and pronunciation. In more severe cases, it can cause high mobility and detachment of the teeth due to alveolar bone absorption. All of these effects lead to the deterioration of the patient’s quality of life (QOL). More than 20 drugs are associated with DIGH [1], principal among them are immunosuppressants, calcium channel blockers, and anticonvulsants [1]. Dongali-Bagtzoglow [1] reported that >70, 6–15, and 50% of DIGH incidences were observed with cyclosporine (CsA; an immunosuppressant), nifedipine (a calcium channel blocker), and phenytoin (an anticonvulsant), respectively.

Since DIGH is a rare adverse event, epidemiologic research is difficult to perform. Spontaneous reporting systems (SRSs) are useful for the detection of rare adverse events and have been recognized as primary tools for pharmacovigilance that reflect the realities of clinical practice. The main aim of regulatory authorities is to collect and store safety reports for monitoring community health. Several pharmacovigilance indexes including the reporting odds ratio (ROR), were developed to evaluate drug-associated adverse events determined though SRS data. The concept of disproportionate analysis of ROR is common in the conventional analysis of SRSs, which attempts to quantify the degree of “unexpectedness” of a drug to adverse event association.

Recently, analysis of time-to-onset data has been proposed as a new method to detect signals for adverse events in SRS. To the best of our knowledge, analyses of the time-to-onset for DIGH using the Japanese Adverse Drug Event Report (JADER) database are rare. Association rule mining has been proposed as an analytical approach in order to study rare adverse drug events, and is a well-established method for discovering undetected relationships such as possible risk factors between variables in huge databases [2–4]. We examined DIGH using both analytical methods, and adjusted for the influence of demography and polypharmacy. This is the first study to evaluate the association between drugs and DIGH using ROR, time-to-onset analysis, and association rule mining. The aims of the study were to obtain new information of risk comparison on drugs or undetected several clinical factor combination, and onset profiles of DIGH for prescription drugs in the real world.

## Methods

### Data sources

The US Food and Drug Administration (FDA) adverse event reporting system (FAERS) is an SRS and the largest and best-known database worldwide. The regulatory authority in Japan, the Pharmaceuticals and Medical Devices Agency (PMDA), controls the SRS of the JADER database. Adverse events recorded in the FAERS database from January 2004 to June 2014 were downloaded from the FDA website (http://www.fda.gov). Relevant information from the JADER database from April 2004 to November 2016 was downloaded from the PMDA website (http://www.pmda.go.jp). We constructed a database that integrated each FAERS and JADER dataset using the FileMaker Pro 13 (FileMaker Inc.). For duplicate entries, we followed the FDA recommendation (http://www.fda.gov/Drugs/GuidanceComplianceRegulatoryInformation/Surveillance/AdverseDrugEffects), and adopted the most recent case number to identify duplicate patient reports and excluded them from the analysis.

We analyzed four immunosuppressants (CsA, everolimus, sirolimus, and mycophenolate mofetil), four calcium channel blockers (amlodipine, benidipine, nicardipine and nifedipine), and 11 anticonvulsants (carbamazepine, clobazam, diazepam, gabapentin, levetiracetam, phenobarbital, phenytoin, primidone, topiramate, valproic acid, and zonisamide). For drug definitions, we used both the general and brand names based on the DrugBank 3.0 and 4.0 (Table 1). Drugs in the FAERS were classified into four categories: Primary Suspect drug (PS), Secondary Suspect drug (SS), Concomitant (C), and Interacting (I); according to their anticipated degree of involvement in adverse events. The analysis was restricted to reports where drugs were recorded as PS and SS in the FAERS database. In the “drug information” table of the JADER database, each drug was assigned a code according to its association with adverse drug reactions: “suspected drug,” “concomitant drug,” or “interacting drug.” The analysis was restricted to reports where drugs were recorded as “suspected drugs” in the JADER database.

**Table 1:** Brand names of drugs

Generic name^a^	Number of brand name	Brand name
Immunosuppressants		
Cyclosporine	6	Gengraf, Neoral, etc.
Everolimus	1	Certican
Sirolimus	1	Rapamune
Mycophenolate mofetil	2	Cellcept, Mucoloc
Calcium Channel Blockers		
Amlodipine	7	Amlocard, Amlodis, etc.
Benidipine^b^	-	-
Nicardipine	3	Cardene, Cardene IV, etc.
Nifedipine	106	Adalat, Adalat 10, etc.
Anticonvulsants		
Carbamazepine	29	Apo-Carbamazepine, Atretol, etc.
Clobazam	6	Chlorepin, Clorepin, etc.
Diazepam	116	Alboral, Aliseum, etc.
Gabapentin	3	Aclonium, Neurontin, etc.
Levetiracetam	1	Keppra
Phenobarbital	138	Adonal, Aephenal, etc.
Phenytoin	130	Aleviatin, Antisacer, etc.
Primidone	36	Apo-Primidone, Cyral, etc.
Topiramate	2	Topamax, Topamax Sprinkle, etc.
Valproic acid	25	Alti-Valproic, Avugane, etc.
Zonisamide	4	Excegran, Exegram, etc.

^a^ Generic name and brand name were used in this analysis ^b^ Benidipine exists only generic name

### Definition of DIGH

The adverse event definitions used in FAERS were those provided by the Medical Dictionary for Regulatory Activities (MedDRA) version 17.1. For the extraction of cases from the FAERS database, we used two preferred terms (PTs), gingival hyperplasia (PT code: 10018283) and gingival hypertrophy (PT code: 10018284). The adverse event definitions used in JADER were those provided by MedDRA version 19.0. In the MedDRA 19.0, the two PTs related to DIGH were combined into “gingival hypertrophy (PT code: 10018284).” Thus, for the extraction of cases from the JADER database, we used the PT gingival hypertrophy (PT code: 10018284).

### Data mining

#### ROR

For the detection of DIGH, we calculated the ROR as the ratio of the odds of reporting a DIGH adverse event versus all other events for a given drug, compared to the reporting odds for all other drugs. We detected the signals when the ROR estimated and lower limits of the corresponding 95% confidence interval (CI) were greater than 1, and at least 2 cases were required to define the signal [5, 6].

#### Time-to-onset analysis

Median, quartile, and Weibull shape parameter (WSP) tests were used to evaluate the time-to-onset analysis [7–10]. We analyzed the time the specific adverse event occurred from when the prescription of specific drugs commenced by using the Weibull distribution parameter. We excluded reports that did not have complete adverse event occurrence and prescription start times. The scale parameter α determined the scale of the distribution function while the shape parameter β determined the shape of the distribution function. In the analysis of the SRSs, the shape parameter β of the Weibull distribution was used to indicate the hazard without reference populations as follows: If the 95% CI of β included 1, the hazard was estimated to be constant over time (random failure type). If the lower limit of the 95% CI of β was greater than 1, the hazard was considered to increase over time (wear-out failure type). If the upper limit of the 95% CI of β was less than 1, the hazard was considered to decrease over time (initial failure type) [11]. The time-to-onset analysis was performed using JMP version 11.0 software (SAS Institute, Cary, NC, USA).

### Association rule mining

The association rule mining approach attempts to evaluate frequent items in databases. Given a set of transactions ***T***, an association rule can be expressed as X → Y, where X and Y are mutually exclusive sets of items [12–14]. The rule’s statistical significance and strength are measured as *support* and *confidence*. The *support* is defined as the percentage of transactions in the data that contain all items in both the antecedent (left hand side) and the consequent (right hand side) of the rule [12–14]. The *support* indicates how frequently the rule occurs in the transaction and has the following formula:

$$ Support=\mathrm{P}\left(\mathrm{X}\cap \mathrm{Y}\right)=\left\{\mathrm{X}\cap \mathrm{Y}\right\}/\left\{\mathrm{D}\right\} $$

where D is total number of transactions in the database. The *confidence* corresponds to the conditional probability P (Y|X). It is important for a rule to have a high *confidence* because it provides an accurate prediction of the association of the items in the rule. The formula for calculating *confidence* is as follows:

$$ Confidence=\mathrm{P}\left(\mathrm{X}\cap \mathrm{Y}\right)/\mathrm{P}\left(\mathrm{X}\right) $$

*Lift* is the probability of X and Y occurring together divided by the multiple of the two individual probabilities for X and Y; that is,

$$ Lift=\mathrm{P}\ \left(\mathrm{X}\cap \mathrm{Y}\right)/\mathrm{P}\left(\mathrm{X}\right)\mathrm{P}\left(\mathrm{Y}\right) $$

Since P (Y) appears in the denominator of the *lift* equation, the *lift* can be considered to be the *confidence* divided by P (Y). The *lift* can be evaluated as follows: *lift* = 1, > 1, and <1 if X and Y are independent, positively correlated, and negatively correlated, respectively. We performed these analyses using the *apriori* function of the *arules* library in the *arules* package R version 3.3.2 software [15].

## Results

The FAERS database contained 5,821,716 reports that were submitted between January 2004 and June 2014. After deleting the duplicate reports, 4,551,642 reports were analyzed. The number of case reports and the RORs are summarized in Table 2. The RORs for cases involving CsA, everolimus, sirolimus, mycophenolate mofetil, amlodipine, nifedipine, carbamazepine, clobazam, levetiracetam, phenobarbital, phenytoin, primidone, topiramate, and valproic acid as PS or SS were 39.4 (95% CI: 30.3–51.2), 4.2 (1.7–10.0), 6.6 (2.5–17.7), 13.1 (7.2–23.2), 94.8 (80.0–112.9), 57.9 (35.7–94.0), 15.1 (10.3–22.3), 65.4 (33.8–126.7), 6.5 (3.6–11.8), 19.7 (8.8–44.0), 65.4 (52.4–82.9), 56.5 (21.1–151.7), 2.9 (1.1–7.7), and 17.5 (12.6–24.4), respectively.

**Table 2:** Number of reports and the reporting odds ratio for gingival hyperplasia by drugs

Drug		Total				Suspected Drug^a^, ^b^			
		Total	Case	ROR	(95% CI)	Total	Case	ROR	(95% CI)
FAERS		4,551,642	628						
Immunosuppressants									
Cyclosporine		20,578	66	25.9	(20.1–33.5)	12,693	62	39.4	(30.3–51.2)
Everolimus		9292	6	4.7	(2.1–10.5)	8756	5	4.2	(1.7–10.0)
Sirolimus		5607	5	6.5	(2.7–15.7)	4415	4	6.6	(2.5–17.7)
Mycophenolate mofetil		20,212	30	11.3	(7.8–16.3)	6213	11	13.1	(7.2–23.2)
Calcium Channel Blockers									
Amlodipine		96,153	195	20.9	(17.7–24.8)	18,509	174	94.8	(80.0–112.9)
Benidipine		423	0	–		29	0	–	
Nicardipine		1838	1	–^c^		674	1	–^c^	
Nifedipine		18,542	23	9.3	(6.1–14.1)	2202	17	57.9	(35.7–94.0)
Anticonvulsants									
Carbamazepine		24,644	31	9.6	(6.7–13.7)	13,494	27	15.1	(10.3–22.3)
Clobazam		3155	11	25.8	(14.2–46.9)	1020	9	65.4	(33.8–126.7)
Diazepam		36,751	9	1.8	(0.9–3.5)	11,581	3	1.9	(0.6–5.9)
Gabapentin		71,069	11	1.1	(0.6–2.0)	17,372	2	0.8	(0.2–3.3)
Levetiracetam		2630	14	39.6	(23.3–67.4)	12,437	11	6.5	(3.6–11.8)
Phenobarbital		7717	18	17.4	(10.9–27.8)	2235	6	19.7	(8.8–44.0)
Phenytoin		20,522	95	39.5	(31.8–49.2)	10,865	85	65.4	(52.4–82.9)
Primidone		2644	10	27.9	(14.9–52.3)	520	4	56.5	(21.1–151.7)
Topiramate		23,635	7	2.2	(1.0–4.6)	10,066	4	2.9	(1.1–7.7)
Valproic acid		34,487	43	9.6	(7.1–13.1)	16,258	37	17.5	(12.6–24.4)
Zonisamide		4138	4	7.1	(2.6–18.9)	1916	2	1.3	(0.7–2.5)
JADER		430,587	80						
Immunosuppressants									
Cyclosporine		8890	14	10.1	(5.7–17.9)	5711	14	15.8	(8.9–28.2)
Mycophenolate mofetil		5320	3	3.1	(1.0–9.9)	3060	1	–^c^	
Calcium Channel Blockers									
Amlodipine		30,451	18	3.8	(2.3–6.5)	3025	18	41.3	(24.4–69.8)
Benidipine		3655	1	–^c^		310	1	–^c^	
Nicardipine		1755	5	16.3	(6.6–40.4)	302	5	96.6	(38.8–240.5)
Nifedipine		10,350	8	4.5	(2.2–9.4)	954	6	36.7	(15.9–84.6)
Anticonvulsants									
Carbamazepine		7411	18	16.6	(9.8–28.1)	5068	16	21.1	(12.2–36.4)
Clobazam		898	11	77.2	(40.7–146.4)	239	10	268.4	(136.6–527.3)
Diazepam		3831	10	16.0	(8.2–31.0)	733	6	47.9	(20.8–110.5)
Gabapentin		1222	2	9.0	(2.2–36.8)	404	1	–^c^	
Levetiracetam		1801	6	19.4	(8.4–44.6)	1177	5	24.4	(9.9–60.5)
Phenobarbital		2622	12	28.9	(15.6–53.5)	1027	9	53.5	(26.7–107.3)
Phenytoin		3712	27	59.0	(37.1–93.9)	1667	22	98.9	(60.4–161.9)
Primidone		100	4	236.0	(84.7–657.8)	25	4	1078.9	(361.8–3217.5)
Topiramate		536	1	–^c^		258	1	–^c^	
Valproic acid		8185	26	24.9	(15.6–39.8)	2618	20	54.9	(33.0–91.2)
Zonisamide		2492	12	30.5	(16.5–56.3)	1073	8	44.8	(21.5–93.2)

^a^ For FAERS, “Primary Suspect Drug” and “Secondary Suspect Drug” were analyzed

^b^ For JADER, “Higiyaku” was analyzed

^c^ Number of cases <2

The JADER contained 430,587 reports submitted between April 2004 and November 2016. The lower limits of the ROR 95% CI for CsA, amlodipine, nicardipine, nifedipine, carbamazepine, clobazam, diazepam, levetiracetam, phenobarbital, phenytoin, primidone, valproic acid, and zonisamide as suspected drug were all greater than one.

### Time-to-onset

**Fig. 1:**
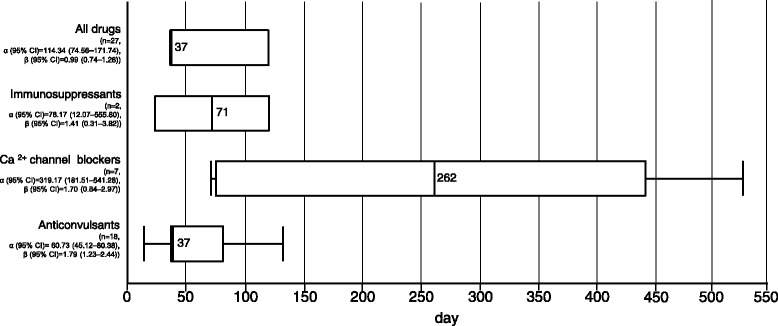
*Box-chart* of time-to-onset analysis for immunosuppressants, calcium channel blockers, and anticonvulsants (the JADER database from April 2004 to November 2016 (*n* = 430,587))

We evaluated data from the JADER database using time-to-onset analysis. The time-to-onset data and WSP are summarized in Fig. 1. The medians and quartile ranges for the onset day of DIGH after treatment with immunosuppressants, calcium channel blockers, and anticonvulsants were 71 (interquartile ranges: 22–120), 262 (76–442), and 37 (37–77) days, respectively. This time-to-onset profile shows that over 50% of DIGH were observed after 37–120 days. The WSP β and 95% CI of immunosuppressants, calcium channel blockers, and anticonvulsants were 1.41 (0.31–3.82), 1.70 (0.84–2.97), and 1.79 (1.23–2.44), respectively (Fig. 1). The WSP β and 95% CI lower limit of anticonvulsants exceeded 1, which describes a wear-out failure type, indicating a significant association between anticonvulsants and DIGH.

### Association rule mining

**Fig. 2:**
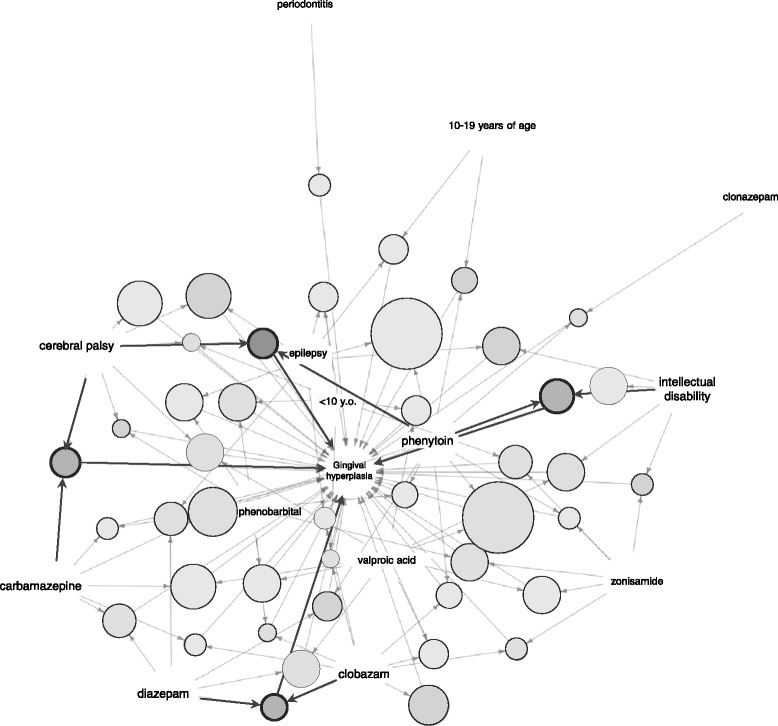
Association rules for gingival hyperplasia (the JADER database from April 2004 to November 2016 (*n* = 430,587)). The *plot* represents items and *rules* as vertices connected with directed edges. Relation parameters are typically added to the plot as labels on the edges or by varying the *color* or width of the *arrows* indicating the edges

We analyzed the JADER database using an association rule mining technique. Association rule mining was applied to the DIGH data using demographic data including age (22 items: < 10 y.o., child, adolescent, etc.), patient history in the all reported cases (8141 items: intellectual disability, cerebral palsy, epilepsy, etc.), administered drugs listed in the Table 1 (19 items: cyclosporine, everolimus, silorimus, etc.), and adverse event [1 item: gingival hypertrophy (PT code: 10018284)]. The *apriori* algorithm efficiently extracts sets of adverse events that occur more frequently than the minimum *support* threshold (defined as 0.00001 in this study), and generates sets of adverse events with the minimum *confidence* threshold (defined as 0.01 in this study). Furthermore, the maximum size of mined frequent itemsets (*maxlen*: a parameter in the *arules* package) was restricted to 3. The result of the mining algorithm was a set of 44 rules (Tables 3 and 4). The *support*, *confidence*, and *lift* for each association rule are summarized in Tables 3 and 4; the association rules in descending order of the *support* are shown in Table 3, and in descending order of the *lift* are shown in Table 4. Anticonvulsants, especially phenytoin, demonstrated a high *support* value (Table 3, Fig. 2). The *lift* aspect of the association rules strength for anticonvulsants, especially phenytoin, carbamazepine, clobazam, and diazepam were high. The association rule of {phenytoin, intellectual disability} → {gingival hypertrophy} with high scores for *lift* and *support* were demonstrated (Table 3 (id [18]), Table 4 (id [4]), Fig. 2). For this rule, the values for *support*, *confidence*, and *lift* were 0.000021, 0.07, and 375.08, respectively. The association rule of {clobazam, diazepam} → {gingival hypertrophy} demonstrated high scores for *lift* (Table 4 (id [3]), Fig. 2). The association rule of {phenytoin, cerebral palsy} → {gingival hypertrophy} and {carbamazepine, cerebral palsy} → {gingival hypertrophy} also demonstrated high scores for *lift* (Table 4 (id [1, 2]), Fig. 2).

**Table 3:** Association parameters of rules (sort by support)

id	lhs (left hand side, antecedent)		rhs (right hand side, consequent)	support	confidence	lift
[1]	{epilepsy, phenytoin}	→	{gingival hypertrophy}	0.000044	0.02	85.21
[2]	{valproic acid, phenytoin}	→	{gingival hypertrophy}	0.000044	0.02	125.83
[3]	{carbamazepine, phenytoin}	→	{gingival hypertrophy}	0.000030	0.02	119.21
[4]	{cerebral palsy}	→	{gingival hypertrophy}	0.000028	0.02	82.79
[5]	{epilepsy, cerebral palsy}	→	{gingival hypertrophy}	0.000028	0.05	233.00
[6]	{carbamazepine, valproic acid}	→	{gingival hypertrophy}	0.000028	0.01	55.27
[7]	{clobazam}	→	{gingival hypertrophy}	0.000026	0.01	62.79
[8]	{intellectual disability}	→	{gingival hypertrophy}	0.000023	0.01	60.38
[9]	{valproic acid, cerebral palsy}	→	{gingival hypertrophy}	0.000023	0.05	244.10
[10]	{epilepsy, intellectual disability}	→	{gingival hypertrophy}	0.000023	0.03	130.43
[11]	{valproic acid, intellectual disability}	→	{gingival hypertrophy}	0.000023	0.03	152.11
[12]	{zonisamide, phenobarbital}	→	{gingival hypertrophy}	0.000023	0.04	183.73
[13]	{epilepsy, phenobarbital}	→	{gingival hypertrophy}	0.000023	0.01	73.54
[14]	{valproic acid, phenobarbital}	→	{gingival hypertrophy}	0.000023	0.02	78.38
[15]	{<10 y.o., phenobarbital}	→	{gingival hypertrophy}	0.000023	0.02	115.45
[16]	{zonisamide, valproic acid}	→	{gingival hypertrophy}	0.000023	0.01	70.41
[17]	{diazepam, valproic acid}	→	{gingival hypertrophy}	0.000023	0.02	123.52
[18]	{phenytoin, intellectual disability}	→	{gingival hypertrophy}	0.000021	0.07	375.08
[19]	{<10 y.o., zonisamide}	→	{gingival hypertrophy}	0.000021	0.03	172.14
[20]	{diazepam, epilepsy}	→	{gingival hypertrophy}	0.000021	0.03	137.71
[21]	{carbamazepine, diazepam}	→	{gingival hypertrophy}	0.000021	0.03	142.39
[22]	{carbamazepine, cerebral palsy}	→	{gingival hypertrophy}	0.000019	0.08	394.31
[23]	{clobazam, valproic acid}	→	{gingival hypertrophy}	0.000019	0.01	76.22
[24]	{diazepam, phenytoin}	→	{gingival hypertrophy}	0.000019	0.04	206.07
[25]	{10–19 years of age, epilepsy}	→	{gingival hypertrophy}	0.000019	0.01	52.51
[26]	{<10 y.o., epilepsy}	→	{gingival hypertrophy}	0.000019	0.01	59.78
[27]	{<10 y.o., valproic acid}	→	{gingival hypertrophy}	0.000019	0.01	58.17
[28]	{phenytoin, cerebral palsy}	→	{gingival hypertrophy}	0.000016	0.12	618.66
[29]	{clobazam, phenytoin}	→	{gingival hypertrophy}	0.000016	0.03	153.34
[30]	{clobazam, diazepam}	→	{gingival hypertrophy}	0.000016	0.07	377.71
[31]	{phenytoin, phenobarbital}	→	{gingival hypertrophy}	0.000016	0.01	75.70
[32]	{10–19 years of age, phenytoin}	→	{gingival hypertrophy}	0.000016	0.04	199.35
[33]	{periodontitis}	→	{gingival hypertrophy}	0.000014	0.01	71.69
[34]	{zonisamide, intellectual disability}	→	{gingival hypertrophy}	0.000014	0.05	256.30
[35]	{clobazam, zonisamide}	→	{gingival hypertrophy}	0.000014	0.02	106.42
[36]	{clobazam, epilepsy}	→	{gingival hypertrophy}	0.000014	0.01	61.88
[37]	{carbamazepine, clobazam}	→	{gingival hypertrophy}	0.000014	0.02	97.02
[38]	{carbamazepine, phenobarbital}	→	{gingival hypertrophy}	0.000014	0.02	79.89
[39]	{zonisamide, phenytoin}	→	{gingival hypertrophy}	0.000014	0.02	89.67
[40]	{phenobarbital, cerebral palsy}	→	{gingival hypertrophy}	0.000012	0.04	194.17
[41]	{<10 y.o., cerebral palsy}	→	{gingival hypertrophy}	0.000012	0.02	107.69
[42]	{clobazam, phenobarbital}	→	{gingival hypertrophy}	0.000012	0.03	141.60
[43]	{<10 y.o., clobazam}	→	{gingival hypertrophy}	0.000012	0.02	111.44
[44]	{clonazepam, phenytoin}	→	{gingival hypertrophy}	0.000012	0.02	114.93

**Table 4:** Association parameters of rules (sort by lift)

id	lhs (left hand side, antecedent)		rhs (right hand side, consequent)	support	confidence	lift
[1]	{phenytoin, cerebral palsy}	→	{gingival hypertrophy}	0.000016	0.12	618.66
[2]	{carbamazepine, cerebral palsy}	→	{gingival hypertrophy}	0.000019	0.08	394.31
[3]	{clobazam, diazepam}	→	{gingival hypertrophy}	0.000016	0.07	377.71
[4]	{phenytoin, intellectual disability}	→	{gingival hypertrophy}	0.000021	0.07	375.08
[5]	{zonisamide, intellectual disability}	→	{gingival hypertrophy}	0.000014	0.05	256.30
[6]	{valproic acid, cerebral palsy}	→	{gingival hypertrophy}	0.000023	0.05	244.10
[7]	{epilepsy, cerebral palsy}	→	{gingival hypertrophy}	0.000028	0.05	233.00
[8]	{diazepam, phenytoin}	→	{gingival hypertrophy}	0.000019	0.04	206.07
[9]	{10–19 years of age, phenytoin}	→	{gingival hypertrophy}	0.000016	0.04	199.35
[10]	{phenobarbital, cerebral palsy}	→	{gingival hypertrophy}	0.000012	0.04	194.17
[11]	{zonisamide, phenobarbital}	→	{gingival hypertrophy}	0.000023	0.04	183.73
[12]	{<10 y.o., zonisamide}	→	{gingival hypertrophy}	0.000021	0.03	172.14
[13]	{clobazam, phenytoin}	→	{gingival hypertrophy}	0.000016	0.03	153.34
[14]	{valproic acid, intellectual disability}	→	{gingival hypertrophy}	0.000023	0.03	152.11
[15]	{carbamazepine, diazepam}	→	{gingival hypertrophy}	0.000021	0.03	142.39
[16]	{clobazam, phenobarbital}	→	{gingival hypertrophy}	0.000012	0.03	141.60
[17]	{diazepam, epilepsy}	→	{gingival hypertrophy}	0.000021	0.03	137.71
[18]	{epilepsy, intellectual disability}	→	{gingival hypertrophy}	0.000023	0.03	130.43
[19]	{valproic acid, phenytoin}	→	{gingival hypertrophy}	0.000044	0.02	125.83
[20]	{diazepam, valproic acid}	→	{gingival hypertrophy}	0.000023	0.02	123.52
[21]	{carbamazepine, phenytoin}	→	{gingival hypertrophy}	0.000030	0.02	119.21
[22]	{<10 y.o., phenobarbital}	→	{gingival hypertrophy}	0.000023	0.02	115.45
[23]	{clonazepam, phenytoin}	→	{gingival hypertrophy}	0.000012	0.02	114.93
[24]	{<10 y.o., clobazam}	→	{gingival hypertrophy}	0.000012	0.02	111.44
[25]	{<10 y.o., cerebral palsy}	→	{gingival hypertrophy}	0.000012	0.02	107.69
[26]	{clobazam, zonisamide}	→	{gingival hypertrophy}	0.000014	0.02	106.42
[27]	{carbamazepine, clobazam}	→	{gingival hypertrophy}	0.000014	0.02	97.02
[28]	{zonisamide, phenytoin}	→	{gingival hypertrophy}	0.000014	0.02	89.67
[29]	{epilepsy, phenytoin}	→	{gingival hypertrophy}	0.000044	0.02	85.21
[30]	{cerebral palsy}	→	{gingival hypertrophy}	0.000028	0.02	82.79
[31]	{carbamazepine, phenobarbital}	→	{gingival hypertrophy}	0.000014	0.02	79.89
[32]	{valproic acid, phenobarbital}	→	{gingival hypertrophy}	0.000023	0.02	78.38
[33]	{clobazam, valproic acid}	→	{gingival hypertrophy}	0.000019	0.01	76.22
[34]	{phenytoin, phenobarbital}	→	{gingival hypertrophy}	0.000016	0.01	75.70
[35]	{epilepsy, phenobarbital}	→	{gingival hypertrophy}	0.000023	0.01	73.54
[36]	{periodontitis}	→	{gingival hypertrophy}	0.000014	0.01	71.69
[37]	{zonisamide, valproic acid}	→	{gingival hypertrophy}	0.000023	0.01	70.41
[38]	{clobazam}	→	{gingival hypertrophy}	0.000026	0.01	62.79
[39]	{clobazam, epilepsy}	→	{gingival hypertrophy}	0.000014	0.01	61.88
[40]	{intellectual disability}	→	{gingival hypertrophy}	0.000023	0.01	60.38
[41]	{<10 y.o., epilepsy}	→	{gingival hypertrophy}	0.000019	0.01	59.78
[42]	{<10 y.o., valproic acid}	→	{gingival hypertrophy}	0.000019	0.01	58.17
[43]	{carbamazepine, valproic acid}	→	{gingival hypertrophy}	0.000028	0.01	55.27
[44]	{10–19 years of age, epilepsy}	→	{gingival hypertrophy}	0.000019	0.01	52.51

## Discussion

Our results suggest that adverse-event signals of DIGH were detected for several drugs in the FAERS and JADER databases. The risk of DIGH is stated in the package inserts of amlodipine, phenytoin, and zonisamide in the US, and CsA, mycophenolate mofetil, amlodipine, benidipine, nifedipine, levetiracetam, phenytoin, topiramate, and valproic acid in Japan, which agrees with our results. Furthermore, we detected signals of DIGH for several drugs such as nicardipine, carbamazepine, clobazam, diazepam, phenobarbital, primidone, and zonisamide that have no adverse-event warning stated in their package inserts in Japan. A more detailed analysis focusing on these drugs should be the subject of future investigation.

Our study had some limitations that should be noted. SRSs are subject to numerous biases and confounders. Since the SRSs did not contain control populations, the ROR does not provide sufficient evidence on causality and should be considered exploratory in the context of signal detection [5–7, 16–21]. The time-to-onset analysis using the WSP method allowed the detection of potential adverse events without requiring a control population [22, 23]. For this reason, we examined the time-to-onset of DIGH using the WSP test.

To the best of our knowledge, no time-to-onset analyses of DIGH have been addressed using SRSs. The aim of the time-to-onset analysis was to obtain new information and compare the risks and onset profiles of DIGH for prescription drugs in the real world. The medians of the times-to-onset values for immunosuppressants and anticonvulsants were 71 and 37 days. The WSP β of anticonvulsants was 1.79 (1.23–2.44) and, so, the hazard was considered to increase over time (Fig. 1). DIGH induced by anticonvulsants was likely to be wear-out failure type. According to a report by Seymour et al. [24], phenytoin-induced gingival hyperplasia can occur within 3 months of drug use, which agrees with our results. These results also corresponded with those of previous reports. To alleviate DIGH, early countermeasures must be initiated. The effective treatments for DIGH are drug substitution or withdrawal, good oral hygiene practices such as plaque control [1], and surgical treatment. DIGH induced by anticonvulsants is clinically important because the number of therapies available for epilepsy has increased. Seizure control is the primary goal of epilepsy treatment [25] and therefore it is difficult to withdraw anticonvulsant drugs. Costa et al. [26] have reported that appropriate plaque control and early detection of periodontal disease is difficult, and that care and periodontal disease tend to worsen easily in patients with refractory epilepsy. The analysis results suggest that early monitoring of the gingival tissue following the observation of gingival hyperplasia in patients administered anticonvulsant agents is required to prevent aggravation of the condition.

The median onset of DIGH by calcium channel blockers was 262 days, which differed from those of anticonvulsants and immunosuppressants. Special attention should be paid to the possibility of DIGH occurrence with these drugs, and careful observation is recommended from 2 to 14 months.

The mechanism mediating the pathogenesis of medication-triggered connective tissue responses in the gingiva is still poorly understood. Some hypotheses have suggested the role of factors such as 1) fibroblasts [27–32], 2) inflammatory cytokines [30, 33–36], and 3) matrix metalloproteinase (MMP) synthesis [31]. CsA, nifedipine, and phenytoin promote the modeling of periodontal fibroblasts through the synthesis of gingival fibroblasts or inhibition of the decomposition of gingival fibroblasts [27–31]. Phenytoin may increase the level of translatable collagen mRNA in human gingival fibroblast [32], while CsA, nifedipine, and phenytoin enhance the synthesis of collagenous proteins in vitro [30, 33–36]. In the case of human gingival fibroblasts simultaneously exposed to nifedipine and interleukin-1β [33], an enhancement of collagenous protein synthesis was observed [33]. CsA may cause a decline in the secretion of MMP-1 and an accumulation of collagenous proteins [31]. The differences in these mechanisms may have affected the ROR value or time-to-onset profiles of each drug.

In the association rule mining approach, since the *lift* values of two combined items, {phenytoin, intellectual disability} were high, patients with intellectual disability have a potential risk of DIGH following treatment with anticonvulsants. Therefore, patients with intellectual disabilities should be monitored carefully. The *lift* values of two combined items {diazepam, clobazam}, which were greater than that of one value were also high enough to suggests a strong association. The *support* value was low, and these data suggest that each association was strong, although the expression rates were low. This information suggests that polypharmacy with anticonvulsants may increase the risk of DIGH. Since Harpaz et al. addressed the issue of confounding factors when applying the association rule mining approach, our obtained association rules might be tabulated independently in the future to evaluate the confounding factors related to DIGH [3].

Patients who were administered calcium channel blockers such as nifedipine or amlodipine demonstrated high drug levels in their gingival crevicular fluid and were likely to be exposed to high levels of these drugs [37, 38]. DIGH was observed with CsA treatment in 25–30% and ≥70% of adults and pediatric patients, respectively [1]. Co-administration of medications with CsA increased the risk of CsA-induced gingival hyperplasia [39], although, the relationship between the dosage, duration of therapy, age, and sex is still not clear. Unfortunately, cases reported in the SRS database do not always contain sufficient information on patient background, drug dosage, drug-drug interactions and mode of administration to allow proper evaluation. Considering the causality constraints of the current analysis, further epidemiological studies are recommended.

## Conclusions

This study was the first to evaluate the incidences of DIGH using SRSs. Despite the limitations inherent to SRS, we identified the risk of DIGH induced by anticonvulsants, immunosuppressants, and calcium channel blockers. We demonstrated the potential risk of DIGH following the long-term use of calcium channel blocker for approximately 260 days. The association rule mining results suggest that patients with intellectual disabilities administered phenytoin, should be monitored carefully. We recommend that patients who experience symptoms related to gingival hyperplasia should be closely monitored and advised to adhere to an appropriate care plan for oral hygiene. Finally, it is our hope that these data will update the information available to clinicians and be potentially useful for improving the management of DIGH.

## Abbreviations

C: Concomitant
CI: Confidence Interval
CsA: Cyclosporine
DIGH: Drug-induced gingival hyperplasia
FAERS: US Food and Drug Administration Adverse Event Reporting System
FDA: Food and Drug Administration
I: Interacting
JADER: Japanese Adverse Drug Event Report
MedDRA: Medical Dictionary for Regulatory Activities
MMP: Matrix metalloproteinase
PMDA: The Pharmaceuticals and Medical Devices Agency
PS: Primary Suspect
PT: Preferred Term
QOL: Quality of life
ROR: Reporting Odds Ratio
SRS: Spontaneous Reporting System
SS: Secondary Suspect
WSP: Weibull Shape Parameter
